# Aorto-Oesophageal Fistula Presenting With Massive Upper Gastrointestinal Haemorrhage in Early Childhood: A Case Report

**DOI:** 10.7759/cureus.110933

**Published:** 2026-06-15

**Authors:** Rukhsana Kausar, Shahir Ulazam

**Affiliations:** 1 Paediatrics, Cork University Hospital, Cork, IRL

**Keywords:** aorto-oesophageal fistula, haemorrhagic shock, massive haematemesis, paediatric gastrointestinal bleeding, thoracic endovascular repair

## Abstract

Aorto-oesophageal fistula is an exceptionally rare but life-threatening cause of upper gastrointestinal bleeding in children. Early diagnosis is challenging because of its nonspecific presentation and rapid clinical deterioration. We report the case of a previously healthy young child who presented with massive haematemesis following a short viral prodrome and rapidly developed haemorrhagic shock. Initial laboratory investigations demonstrated severe acute anaemia requiring activation of the massive haemorrhage protocol and blood product resuscitation. Computed tomography imaging revealed abnormal vascular communication between the thoracic aorta and oesophagus consistent with an aorto-oesophageal fistula associated with significant aortic malformation. The patient required urgent multidisciplinary management, including intubation, transfer to a tertiary paediatric intensive care unit, and endovascular repair with covered aortic stent placement. Extensive investigations excluded infectious, inflammatory, and connective tissue disorders. The child recovered well following intervention, was gradually reintroduced to enteral feeding, and was discharged with ongoing specialist follow-up. This case highlights the importance of considering vascular causes in unexplained massive paediatric haematemesis. Prompt cross-sectional imaging, early haemodynamic stabilization, and multidisciplinary intervention are essential for survival. Endovascular repair may provide a life-saving minimally invasive treatment option in selected paediatric patients.

## Introduction

Massive upper gastrointestinal bleeding is rare in children and is most commonly associated with mucosal disease or portal hypertension [1]. Aorto-oesophageal fistula is an exceptionally uncommon but frequently fatal cause of upper gastrointestinal bleeding, predominantly described in adults [2,3]. Paediatric cases are rare and are often associated with congenital aortic abnormalities or infections. Early diagnosis and rapid intervention are essential for survival [2,4].

Here, we discuss the case of a previously healthy child who presented with acute haematemesis following a short viral prodrome and rapidly developed haemorrhagic shock. Cross-sectional imaging revealed abnormal vascular communication between the thoracic aorta and oesophagus, consistent with an aorto-oesophageal fistula in the context of a significant aortic malformation. This case highlights a rare but life-threatening cause of paediatric upper gastrointestinal bleeding and emphasizes the importance of early recognition and prompt multidisciplinary intervention.

## Case presentation

A previously healthy 12-month-old female child presented with a 24-hour history of viral symptoms, including fever up to 40°C, followed by two episodes of large-volume haematemesis with clots. There was no history of trauma, foreign body ingestion, bleeding tendency, or previous hospital admission. The family history was unremarkable.

On arrival, the patient was haemodynamically unstable and received a bolus of Hartmann’s solution. Empirical treatment with cefotaxime, vitamin K, tranexamic acid, and pantoprazole was commenced. Examination revealed a well-grown child with a soft, non-tender abdomen and normal cardiovascular and respiratory findings.

Initial laboratory tests showed haemoglobin at 8.8 g/dL (Table 1), falling to 5.9 g/dL within four hours following further haematemesis. Massive haemorrhage protocol was activated, and the child received two units of red cell concentrate, two units of plasma, and one unit of platelets.

**Table 1:** Laboratory investigations at presentation CRP: C-reactive protein; INR: international normalised ratio; PT: prothrombin time; aPTT: activated partial thromboplastin time

Parameters	Patient Value	Reference Range
Haemoglobin	8.8 g/dL (5.9 g/dL)	11.5–15.5 g/dL
White Cell Count	9.6×10⁹/L	4.5–13.5 ×10⁹/L
Platelets	268 ×10⁹/L	150–450 ×10⁹/L
CRP	10.7 mg/L	<5 mg/L
INR	1.2	0.9–1.2
PT	12.6 seconds	11–15 seconds
aPTT	24 seconds	25–35 seconds
Fibrinogen	2.0 g/L	1.5–4.0 g/L
Urea	11.1 mmol/L	2.5–7.8 mmol/L
Creatinine	25 µmol/L	Age dependent
Lactate	2.5 mmol/L	0.5–2.2 mmol/L

Computed tomography (CT) of the chest and upper gastrointestinal tract demonstrated vascular communication between the thoracic aorta and oesophagus at the level of T7, consistent with an aorto-oesophageal fistula, in the context of severe aortic malformation. Axial CT images demonstrated the abnormal relationship between the thoracic aorta and oesophagus and associated mediastinal abnormalities (Figures 1, 2). Cross-sectional CT imaging is considered critical for rapid diagnosis in suspected aorto-oesophageal fistula [2,4,5].

**Figure 1:**
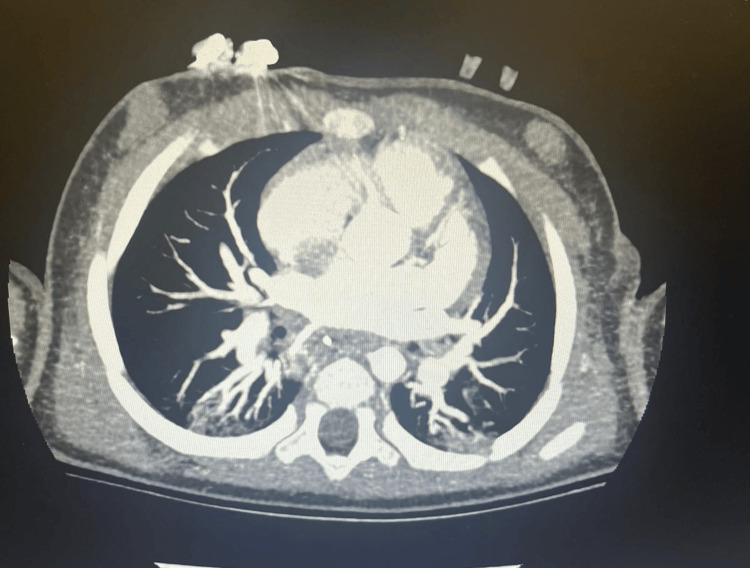
Contrast-enhanced CT demonstrating communication between the thoracic aorta and oesophagus, consistent with aorto-oesophageal fistula.

**Figure 2:**
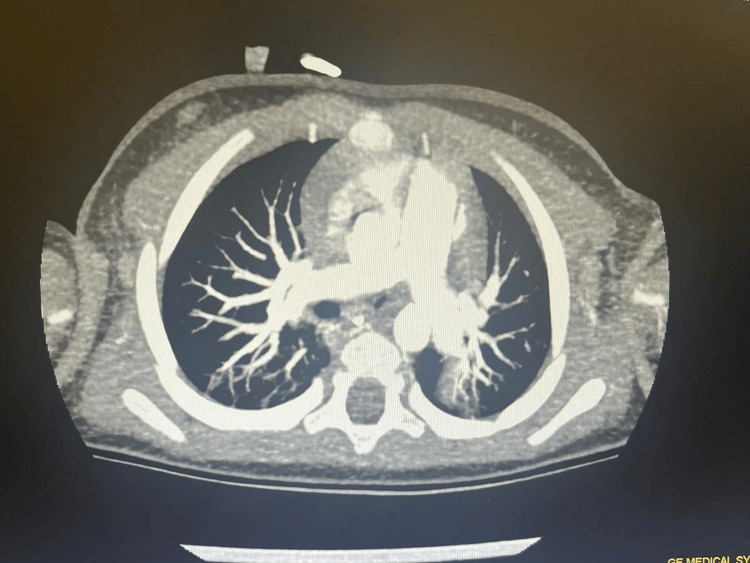
Axial CT image demonstrating thoracic aortic abnormality and adjacent mediastinal changes associated with the aorto-oesophageal fistula prior to endovascular repair.

The child was intubated for haemodynamic stabilization following multidisciplinary input and transferred to a tertiary paediatric intensive care unit. The patient's young age and critical haemodynamic status made immediate open surgical repair technically challenging and high risk. Endovascular stent placement was therefore used as a life-saving strategy to achieve prompt exclusion of the fistula and stabilization of the patient. Endovascular repair was performed with implantation of a covered aortic stent via the left carotid artery (Figures 3, 4).

**Figure 3:**
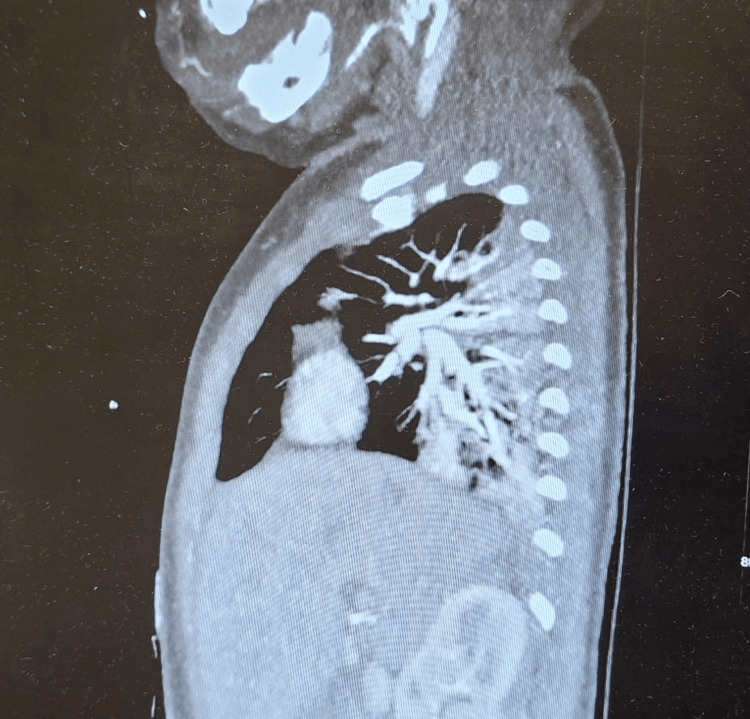
Endovascular repair demonstrating successful deployment of a covered thoracic aortic stent with exclusion of the fistulous communication.

**Figure 4:**
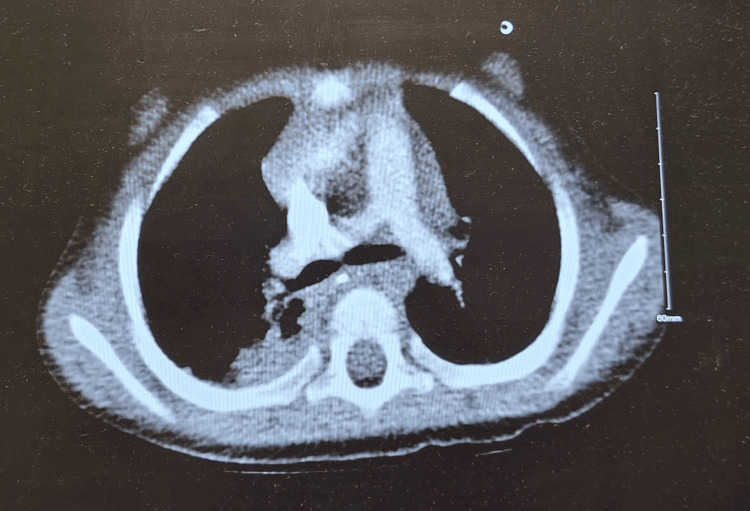
Follow-up CT angiogram confirming successful repair of the aorto-oesophageal fistula

Broad-spectrum antimicrobial therapy was initiated prophylactically because of the high risk of mediastinal contamination and infection associated with an aorto-oesophageal fistula, and enteral feeding was withheld with the commencement of total parenteral nutrition. A cervical lymph node biopsy initially raised concern for *Aspergillus* species, prompting escalation to liposomal amphotericin B. However, subsequent cultures and *Aspergillus *polymerase chain reaction (PCR) testing were negative, and no additional clinical, radiological, or microbiological evidence of invasive fungal infection was identified. Following multidisciplinary review, antifungal therapy was discontinued. Investigations for tuberculosis, vasculitis, and connective tissue disease yielded negative results.

The child was extubated the following day and remained haemodynamically stable. An upper gastrointestinal contrast study performed 10 days later showed no evidence of oesophageal perforation, allowing the gradual reintroduction of enteral feeding. Follow-up imaging confirmed that the stent was in situ with no evidence of extravasation. The child had no neurological deficits and was discharged with multidisciplinary follow-up.

## Discussion

Aorto-esophageal fistula is a rare but catastrophic cause of paediatric upper gastrointestinal bleeding, with high mortality if the diagnosis is delayed [2,3]. Congenital aortic abnormalities are recognized as predisposing factors in children [6], although infectious and inflammatory causes must be excluded. The condition was first described by Dubrueil in 1818 [7], while Chiari later described the classical triad of thoracic pain, sentinel haemorrhage, and fatal exsanguination [8].

This case demonstrates the importance of early cross-sectional imaging in unexplained massive haematemesis and highlights the role of endovascular repair as a life-saving intervention in selected paediatric patients. Endovascular repair offers a potentially life-saving and minimally invasive treatment option in selected paediatric patients [9-11]. Endovascular techniques have increasingly emerged as effective minimally invasive approaches in thoracic aortic emergencies. Current paediatric gastrointestinal endoscopy guidelines emphasize urgent evaluation in severe upper gastrointestinal bleeding [12].

## Conclusions

Aorto-oesophageal fistula is a rare but catastrophic cause of massive upper gastrointestinal bleeding in children and should be considered in cases of unexplained haematemesis with haemodynamic instability. Early recognition, rapid haemodynamic resuscitation, and prompt cross-sectional imaging are critical for diagnosis and survival. This case highlights the importance of multidisciplinary management and demonstrates that endovascular repair can be an effective life-saving treatment option in selected paediatric patients.
